# Supplementary figures and images for: Intergenerational transport of double-stranded RNA in C. elegans can limit heritable epigenetic changes
Source: eLife. 2025 Feb 4;13:RP99149. doi: 10.7554/eLife.99149 (PMC11793870; doi:10.7554/eLife.99149)

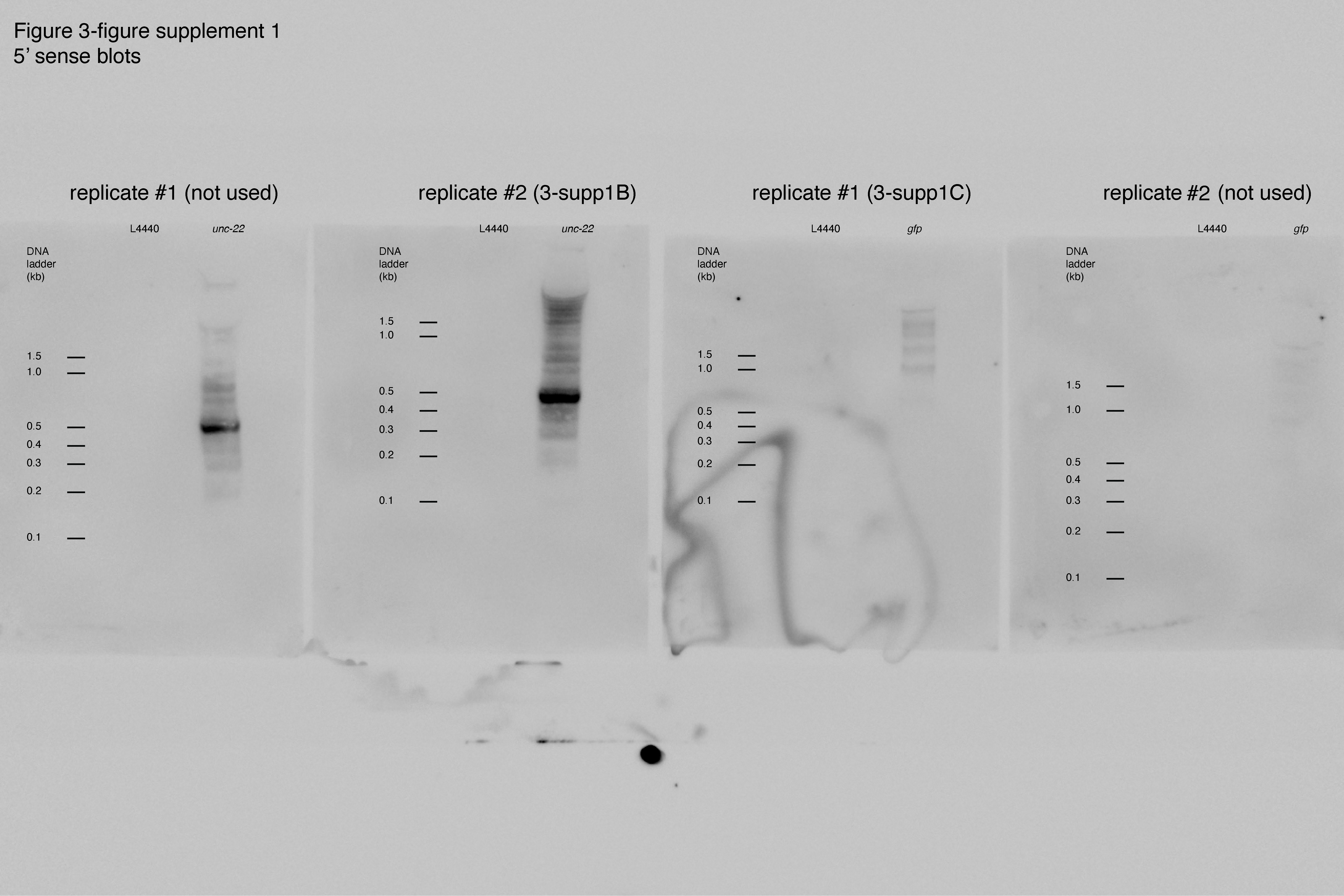

Supplement: Figure 3—figure supplement 1—source data 1. — Raw images are available at https://doi.org/10.6084/m9.figshare.25036142.v1. [file elife-99149-fig3-figsupp1-data1.zip › Figure3_figure_supplement_1/4b_c/20180517_AB404CD404_standard10min.tif]

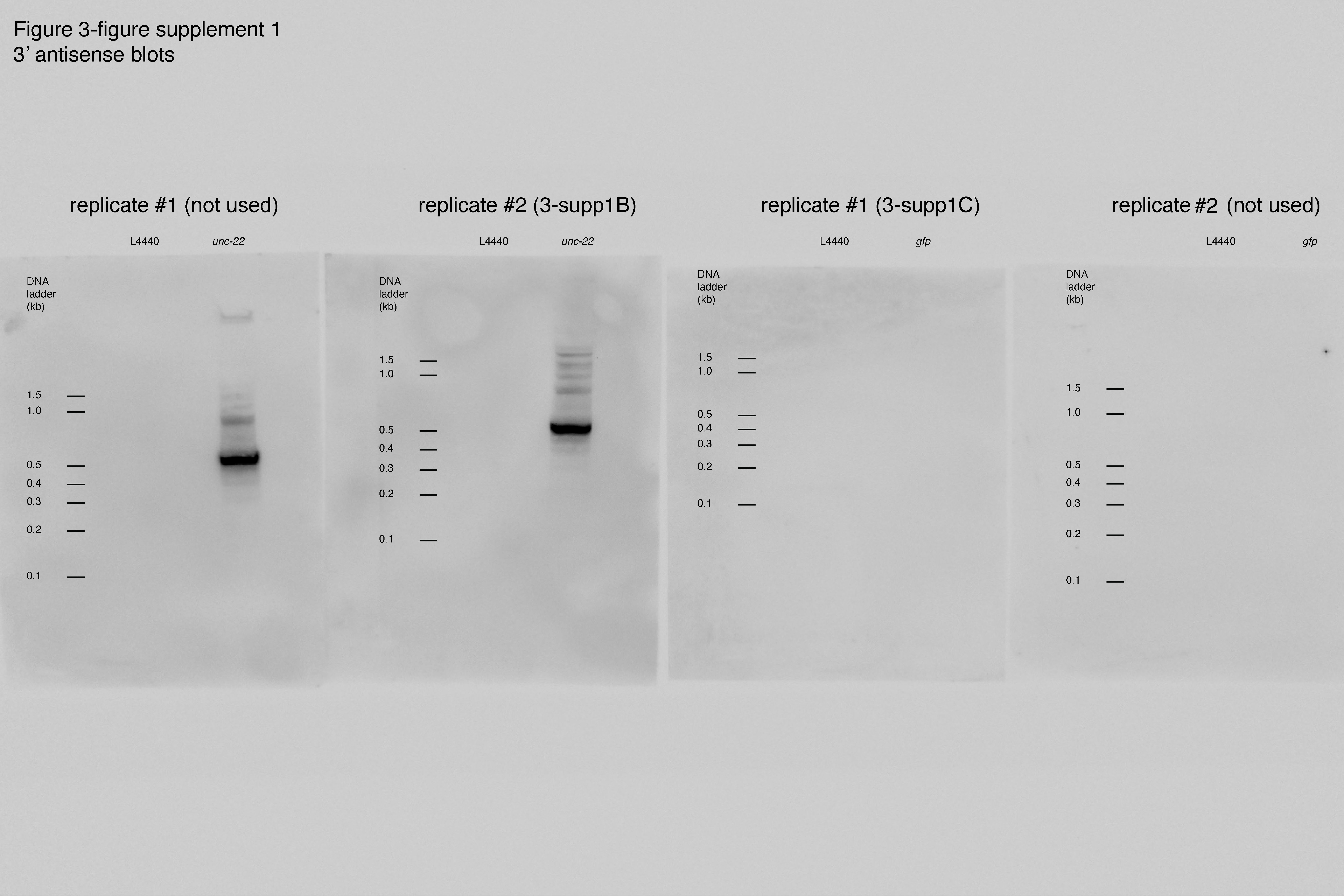

Supplement: Figure 3—figure supplement 1—source data 1. — Raw images are available at https://doi.org/10.6084/m9.figshare.25036142.v1. [file elife-99149-fig3-figsupp1-data1.zip › Figure3_figure_supplement_1/4b_c/20180511_AB401CD401_standard10min.tif]

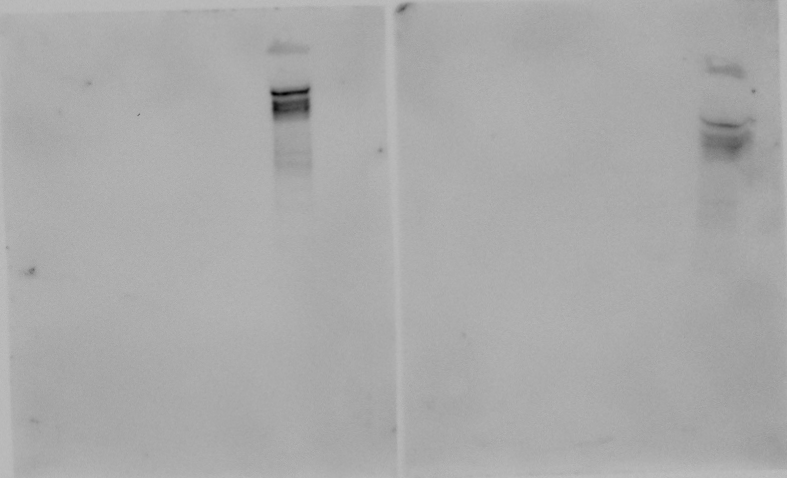

Supplement: Figure 3—figure supplement 1—source data 1. — Raw images are available at https://doi.org/10.6084/m9.figshare.25036142.v1. [file elife-99149-fig3-figsupp1-data1.zip › Figure3_figure_supplement_1/4b_c/20180712_CD405redo_standard10min240lvl_unlabelled.tif]

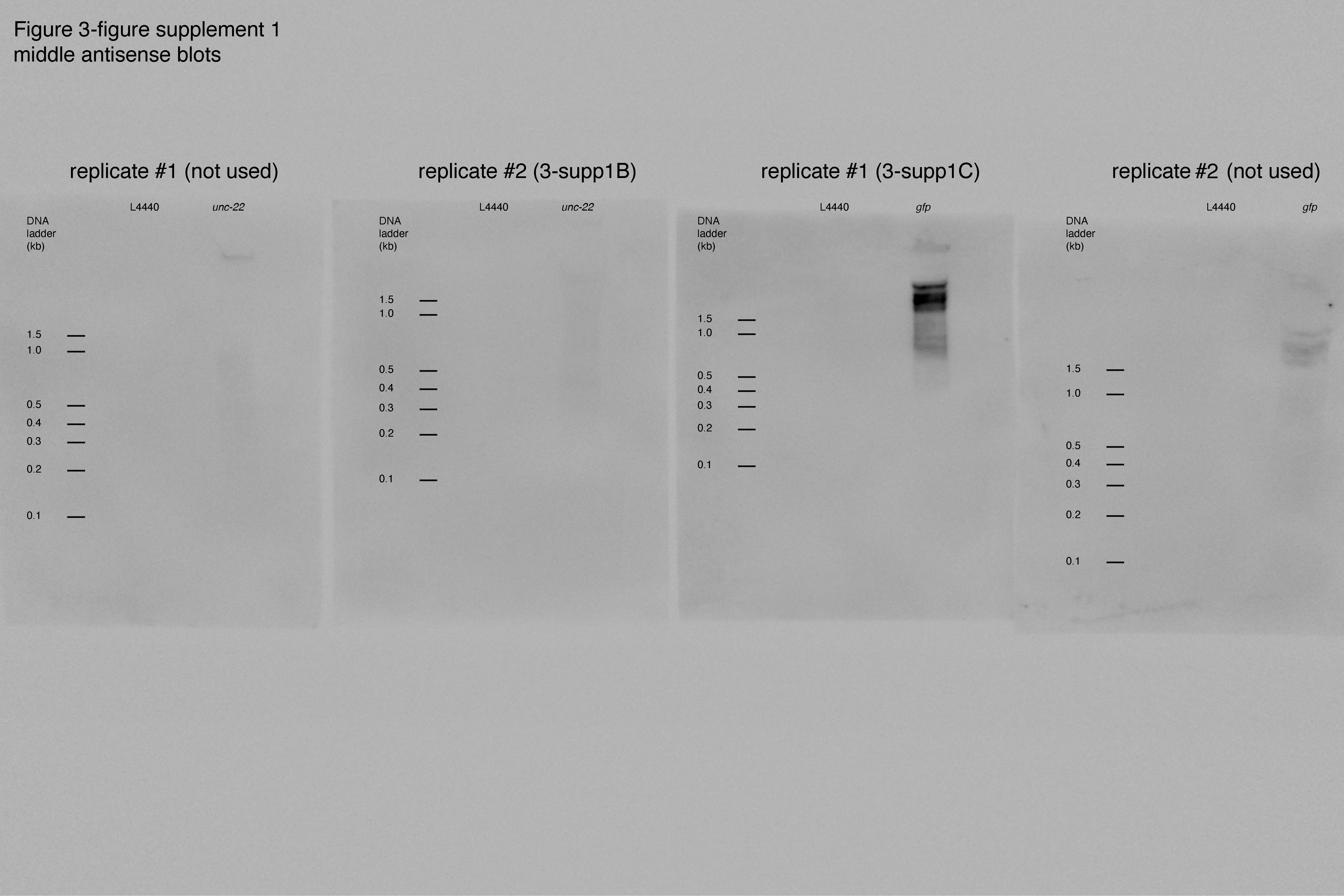

Supplement: Figure 3—figure supplement 1—source data 1. — Raw images are available at https://doi.org/10.6084/m9.figshare.25036142.v1. [file elife-99149-fig3-figsupp1-data1.zip › Figure3_figure_supplement_1/4b_c/20180515_AB402CD402_standard10min.tif]

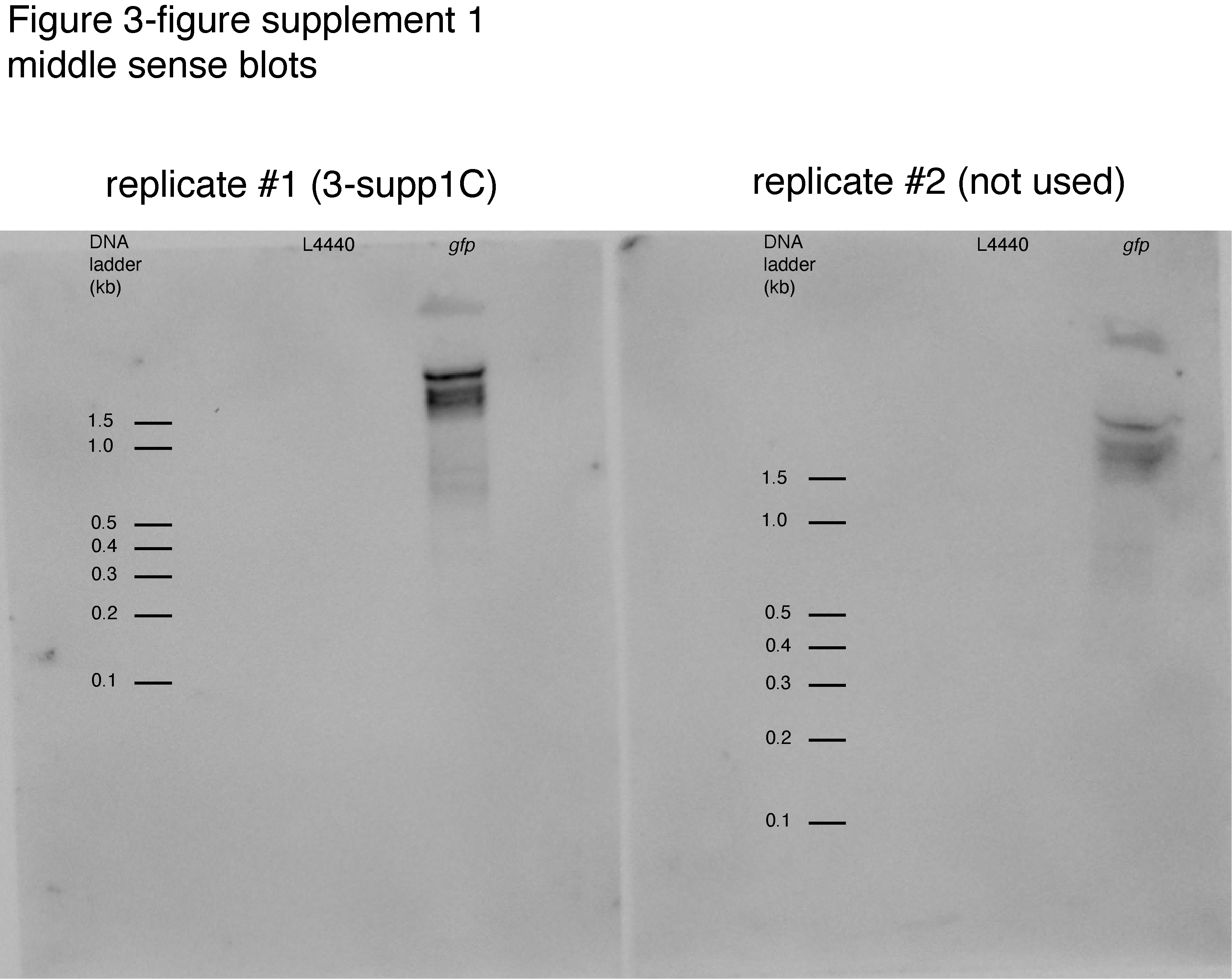

Supplement: Figure 3—figure supplement 1—source data 1. — Raw images are available at https://doi.org/10.6084/m9.figshare.25036142.v1. [file elife-99149-fig3-figsupp1-data1.zip › Figure3_figure_supplement_1/4b_c/20180712_CD405redo_standard10min240lvl.tif]

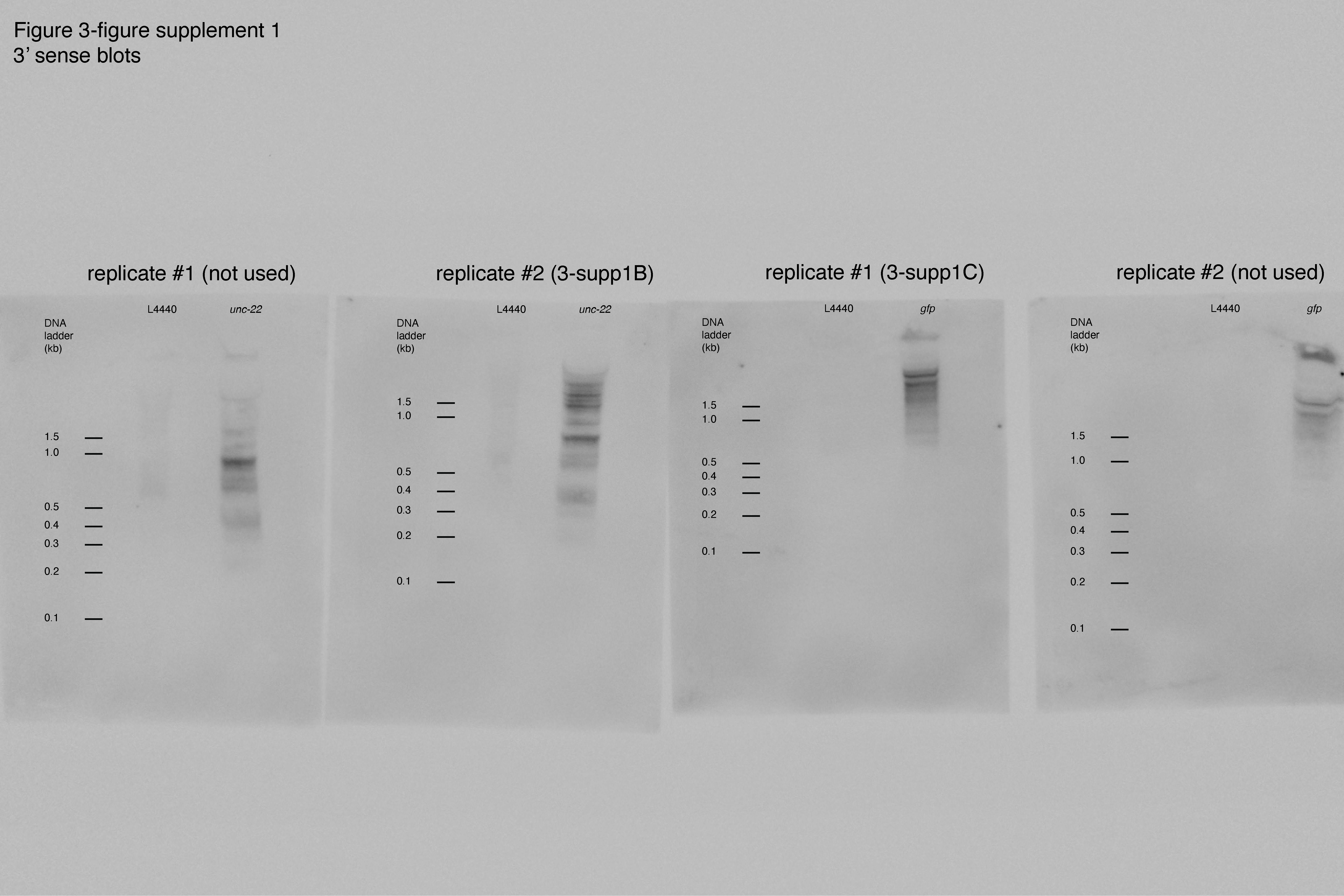

Supplement: Figure 3—figure supplement 1—source data 1. — Raw images are available at https://doi.org/10.6084/m9.figshare.25036142.v1. [file elife-99149-fig3-figsupp1-data1.zip › Figure3_figure_supplement_1/4b_c/20180521_AB406CD406_standard10min.tif]

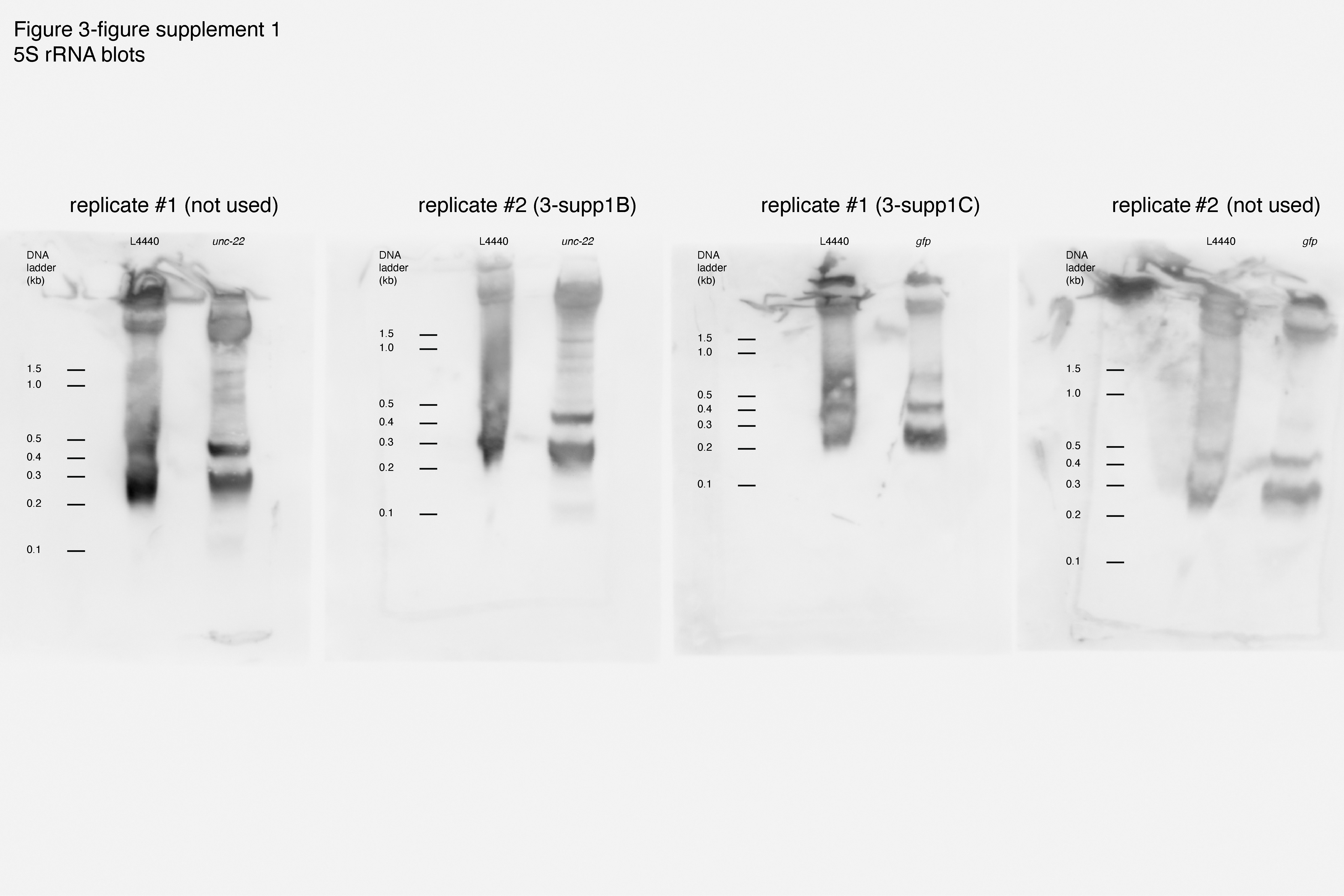

Supplement: Figure 3—figure supplement 1—source data 1. — Raw images are available at https://doi.org/10.6084/m9.figshare.25036142.v1. [file elife-99149-fig3-figsupp1-data1.zip › Figure3_figure_supplement_1/4b_c/20180507_ABCD5S_standard10min.tif]

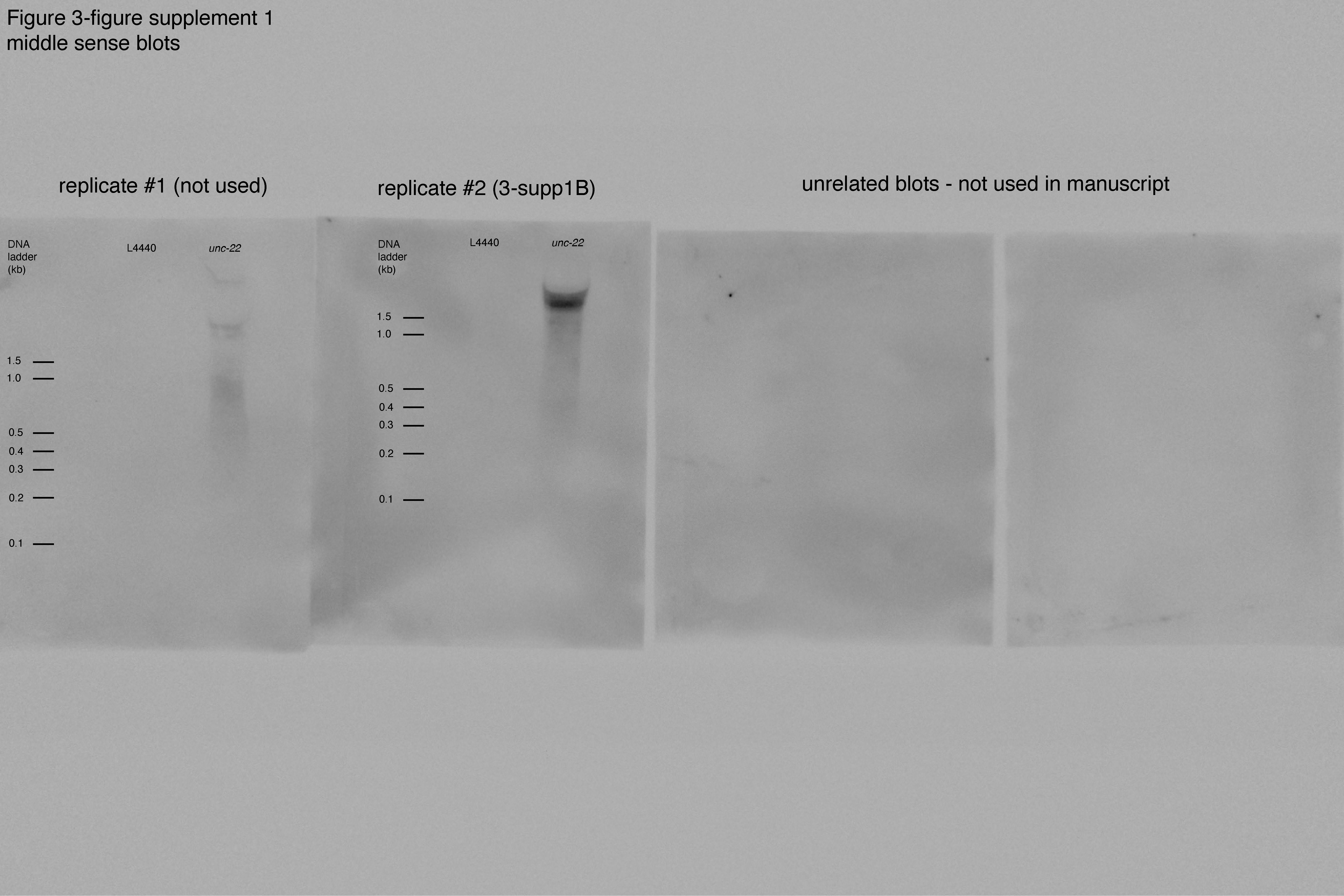

Supplement: Figure 3—figure supplement 1—source data 1. — Raw images are available at https://doi.org/10.6084/m9.figshare.25036142.v1. [file elife-99149-fig3-figsupp1-data1.zip › Figure3_figure_supplement_1/4b_c/20180518_AB405CD405_standard10min.tif]

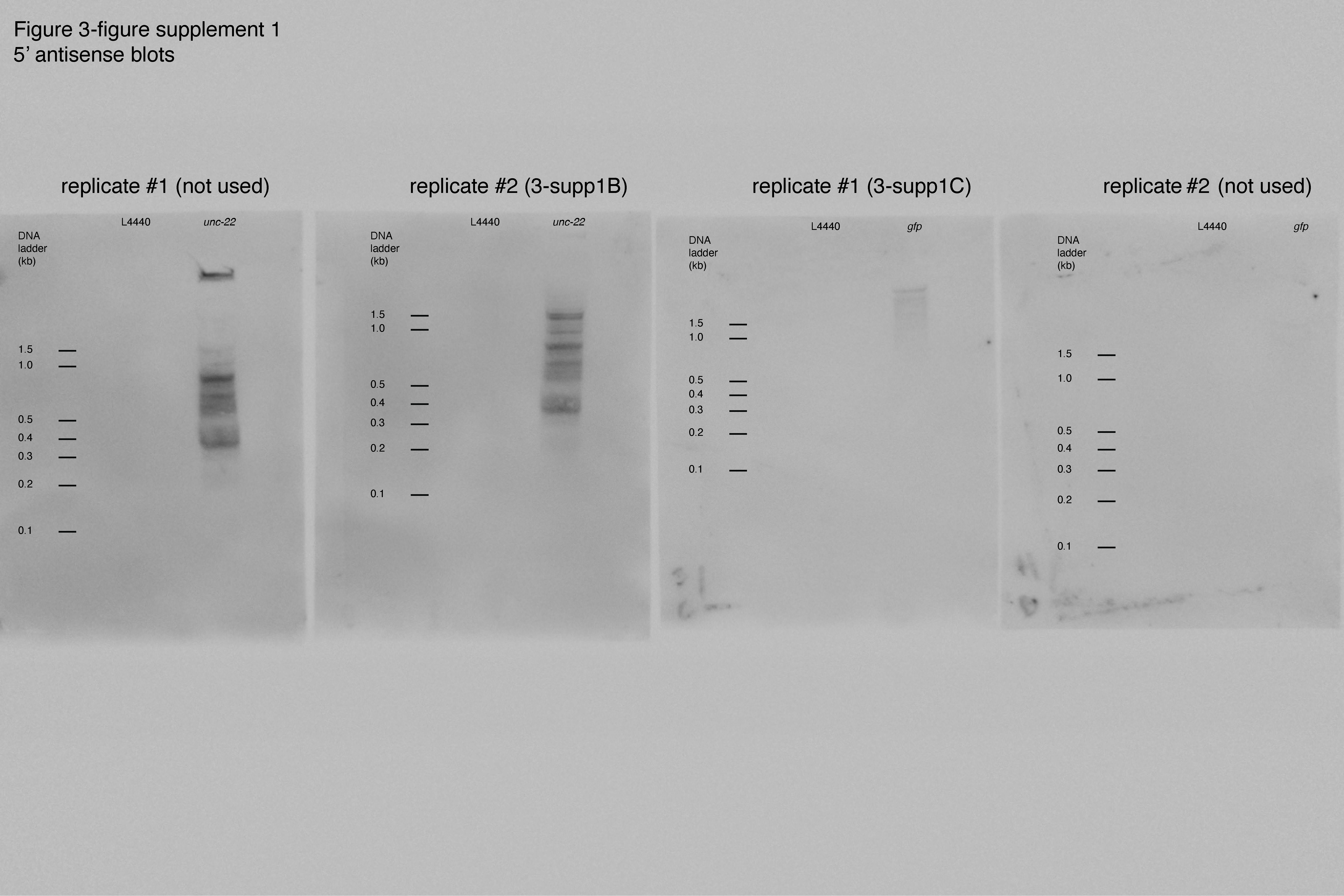

Supplement: Figure 3—figure supplement 1—source data 1. — Raw images are available at https://doi.org/10.6084/m9.figshare.25036142.v1. [file elife-99149-fig3-figsupp1-data1.zip › Figure3_figure_supplement_1/4b_c/20180516_AB403CD403_standard10min.tif]

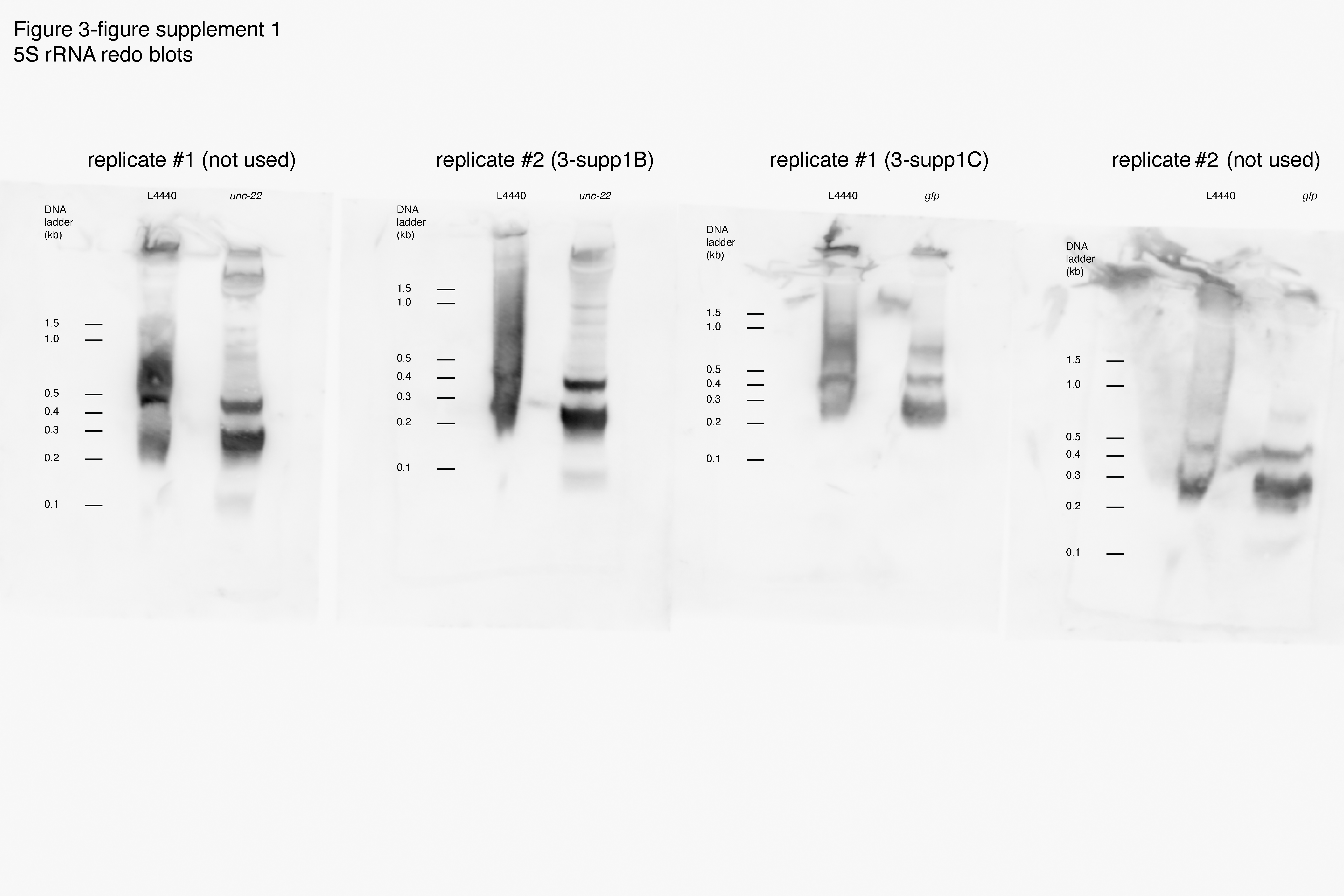

Supplement: Figure 3—figure supplement 1—source data 1. — Raw images are available at https://doi.org/10.6084/m9.figshare.25036142.v1. [file elife-99149-fig3-figsupp1-data1.zip › Figure3_figure_supplement_1/4b_c/20180522_ABCD5Sredo_standard10min.tif]

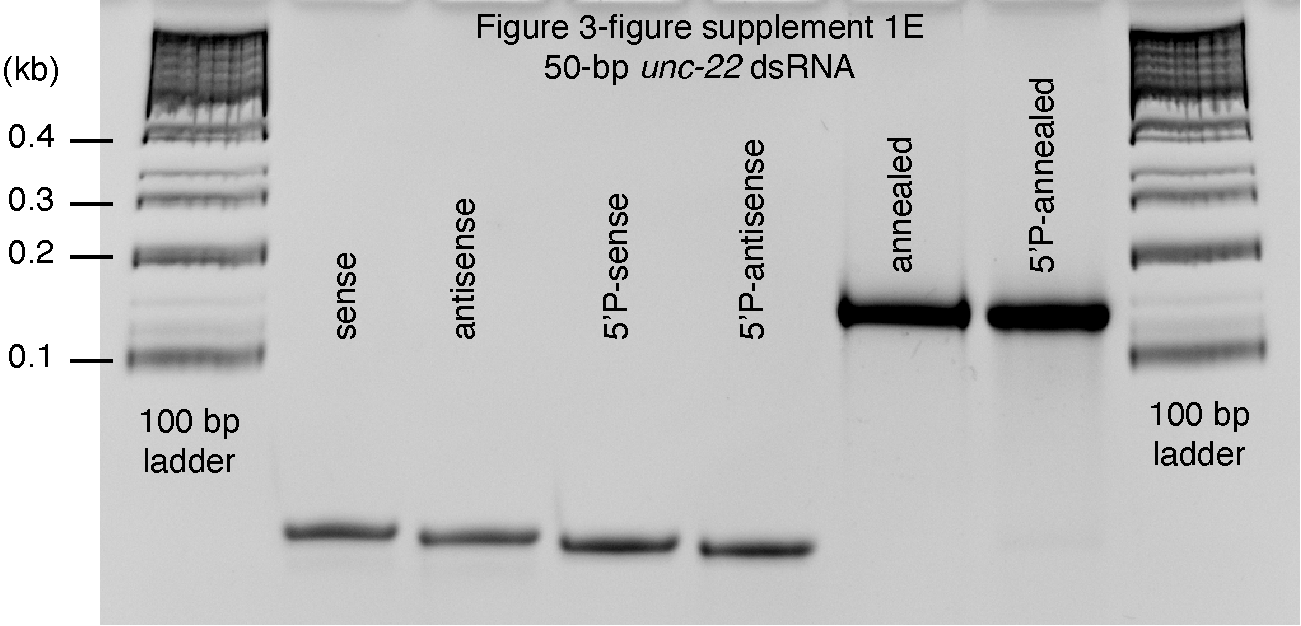

Supplement: Figure 3—figure supplement 1—source data 1. — Raw images are available at https://doi.org/10.6084/m9.figshare.25036142.v1. [file elife-99149-fig3-figsupp1-data1.zip › Figure3_figure_supplement_1/4e/5.31.19unc.tif]

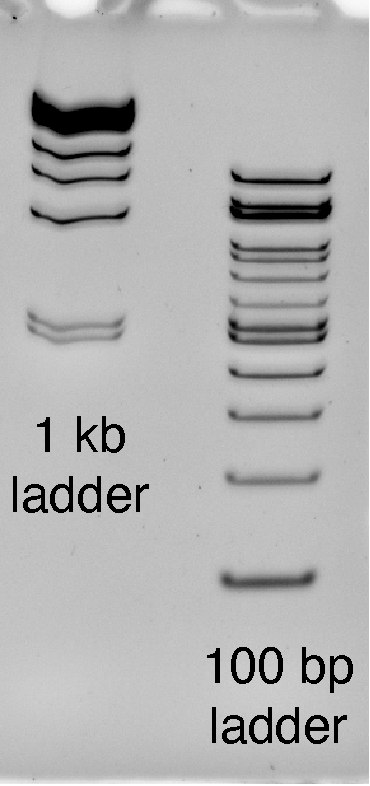

Supplement: Figure 3—figure supplement 1—source data 1. — Raw images are available at https://doi.org/10.6084/m9.figshare.25036142.v1. [file elife-99149-fig3-figsupp1-data1.zip › Figure3_figure_supplement_1/4b_c/ladders/5.3.18A.tif]

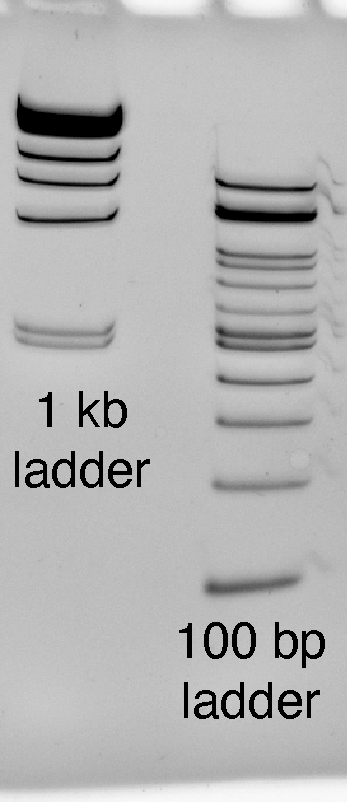

Supplement: Figure 3—figure supplement 1—source data 1. — Raw images are available at https://doi.org/10.6084/m9.figshare.25036142.v1. [file elife-99149-fig3-figsupp1-data1.zip › Figure3_figure_supplement_1/4b_c/ladders/5.3.18B.tif]

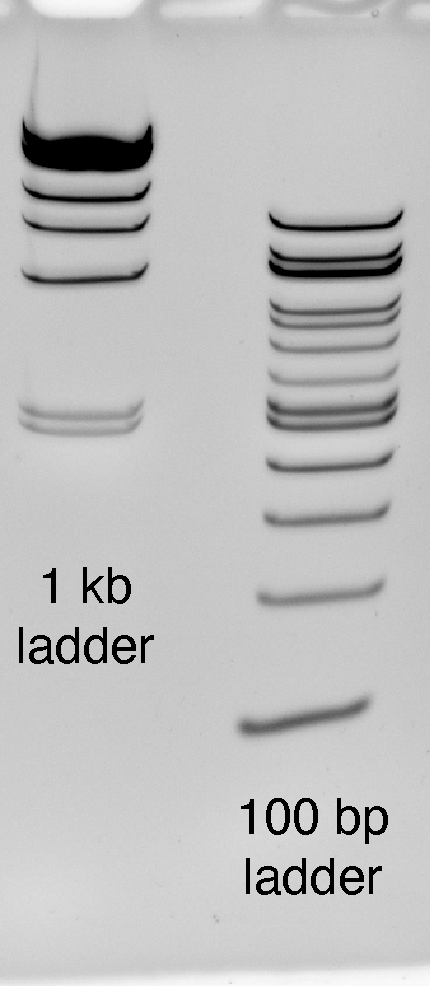

Supplement: Figure 3—figure supplement 1—source data 1. — Raw images are available at https://doi.org/10.6084/m9.figshare.25036142.v1. [file elife-99149-fig3-figsupp1-data1.zip › Figure3_figure_supplement_1/4b_c/ladders/5.4.18C.tif]

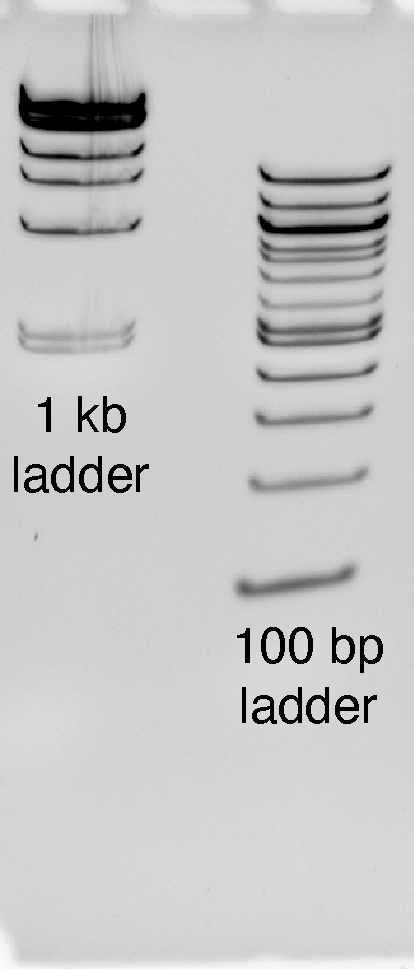

Supplement: Figure 3—figure supplement 1—source data 1. — Raw images are available at https://doi.org/10.6084/m9.figshare.25036142.v1. [file elife-99149-fig3-figsupp1-data1.zip › Figure3_figure_supplement_1/4b_c/ladders/5.4.18D.tif]

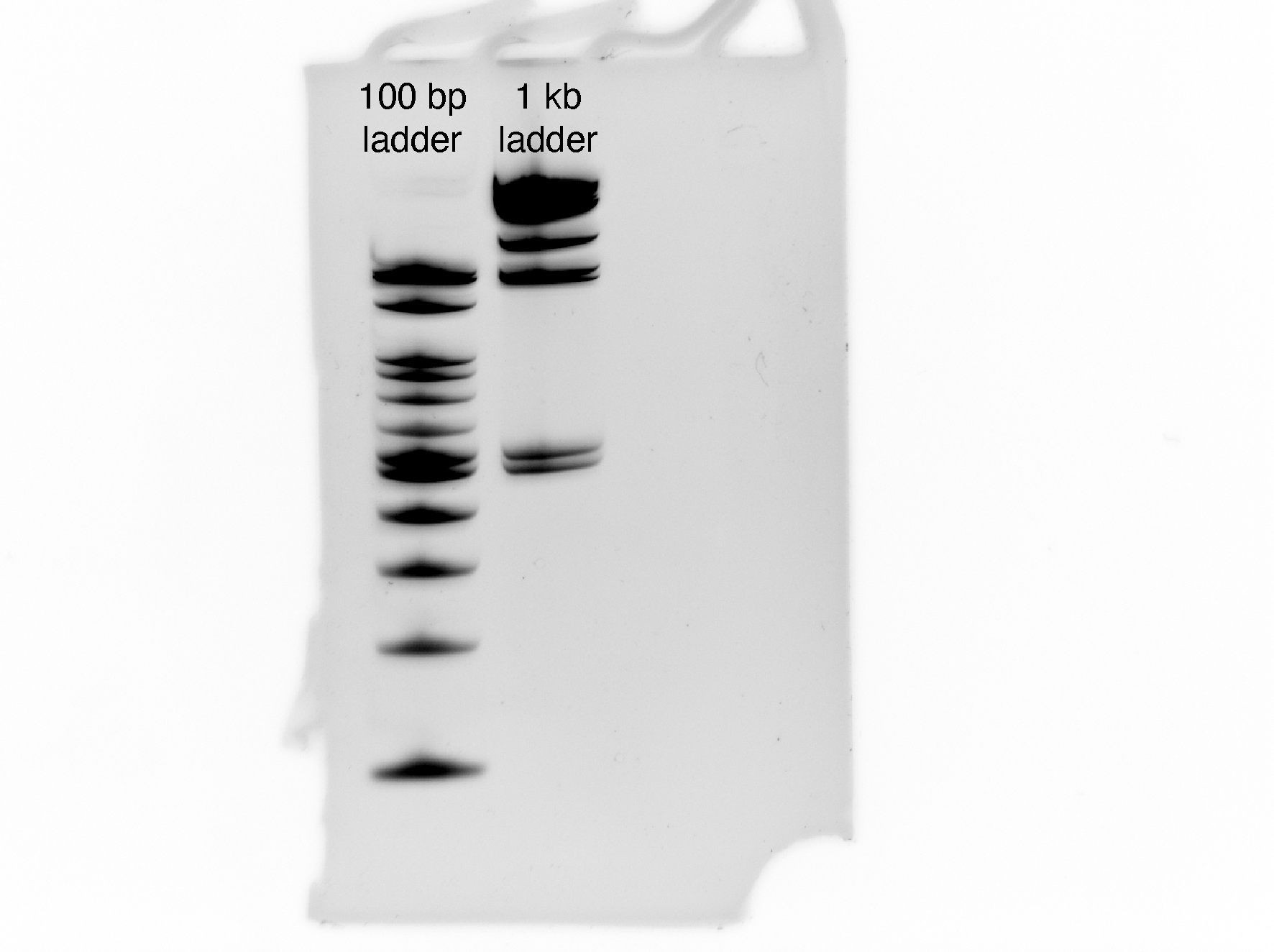

Supplement: Figure 3—figure supplement 1—source data 1. — Raw images are available at https://doi.org/10.6084/m9.figshare.25036142.v1. [file elife-99149-fig3-figsupp1-data1.zip › Figure3_figure_supplement_1/4d/ladders/3.26.19.tif]

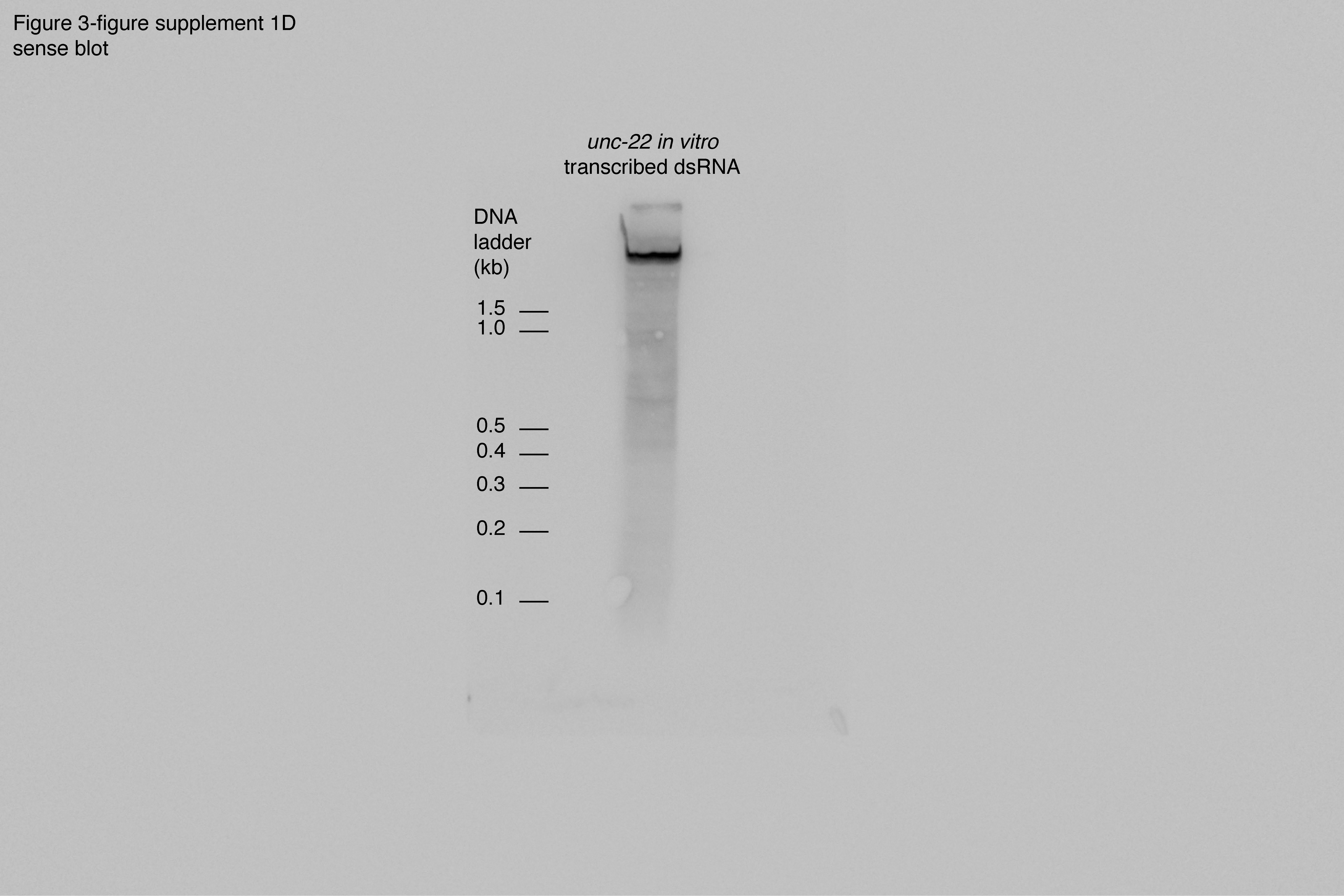

Supplement: Figure 3—figure supplement 1—source data 1. — Raw images are available at https://doi.org/10.6084/m9.figshare.25036142.v1. [file elife-99149-fig3-figsupp1-data1.zip › Figure3_figure_supplement_1/4d/sense/20190328_unc22401_standard5min.tif]

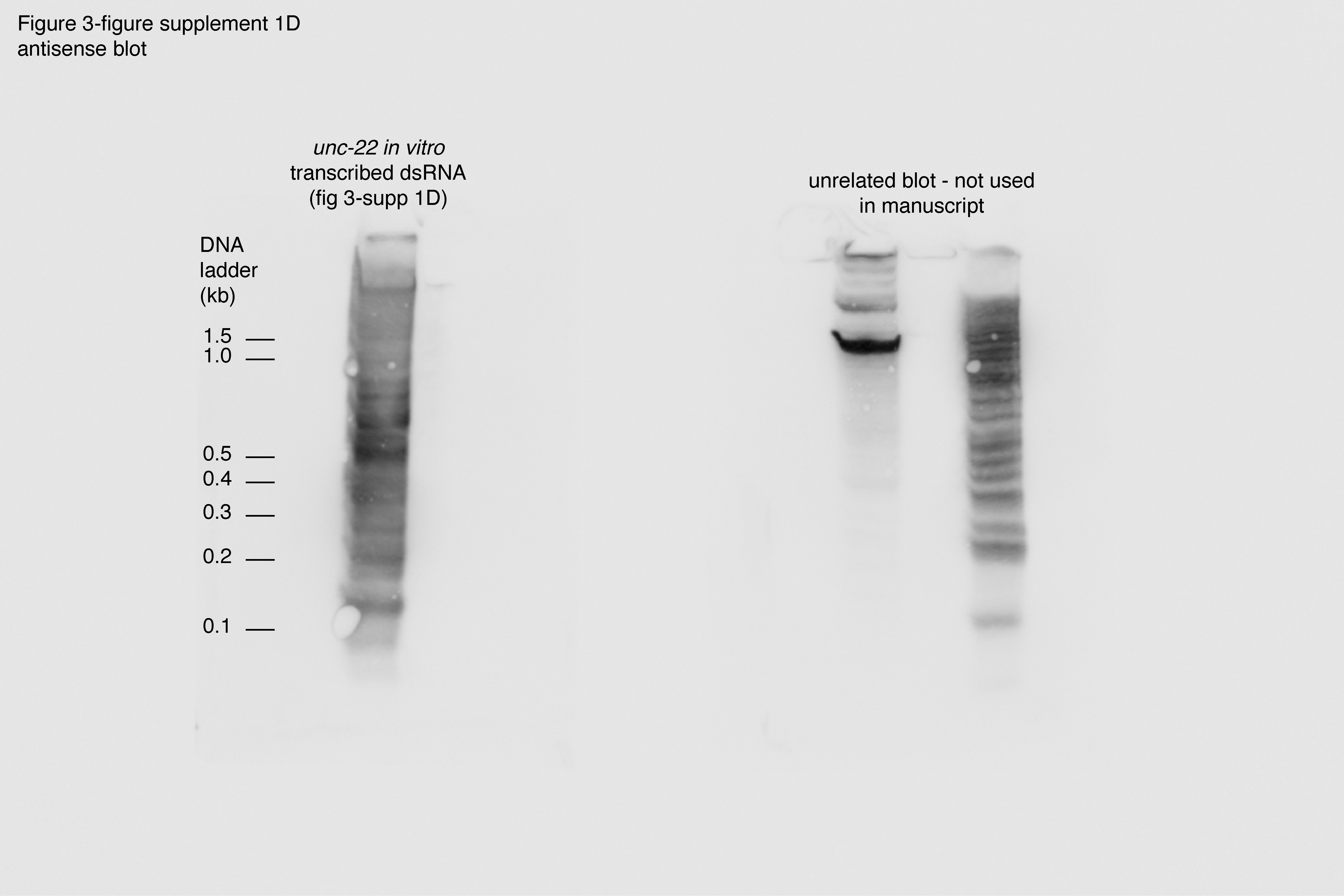

Supplement: Figure 3—figure supplement 1—source data 1. — Raw images are available at https://doi.org/10.6084/m9.figshare.25036142.v1. [file elife-99149-fig3-figsupp1-data1.zip › Figure3_figure_supplement_1/4d/antisense/20190517_unc404_unclacZREV_standard5min.tif]

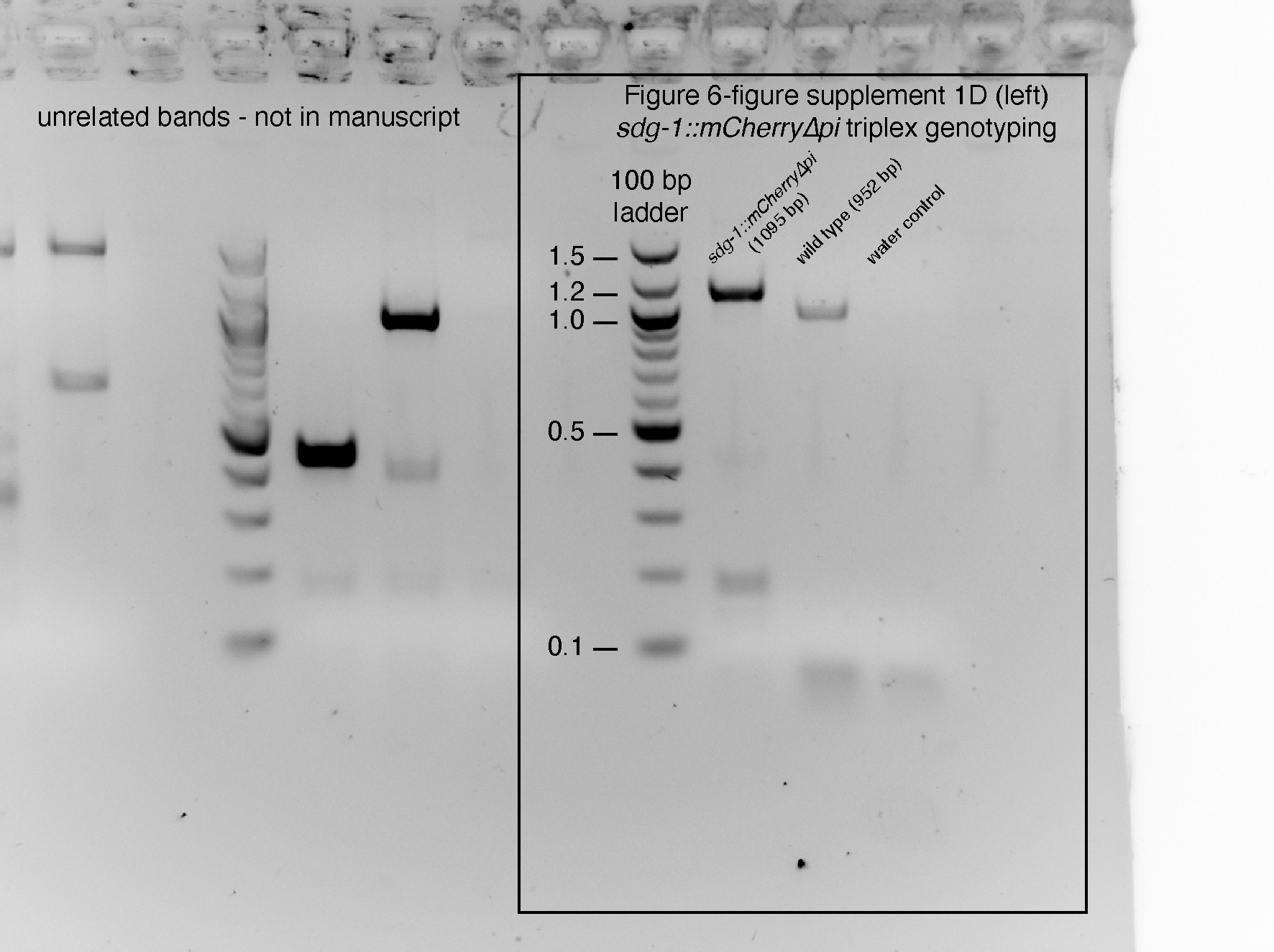

Supplement: Figure 6—figure supplement 1—source data 1. — Raw images are available at https://doi.org/10.6084/m9.figshare.25036142.v1. [file elife-99149-fig6-figsupp1-data1.zip › Figure6_figure_supplement_1/8d/04_12_2021_f07s.tif]

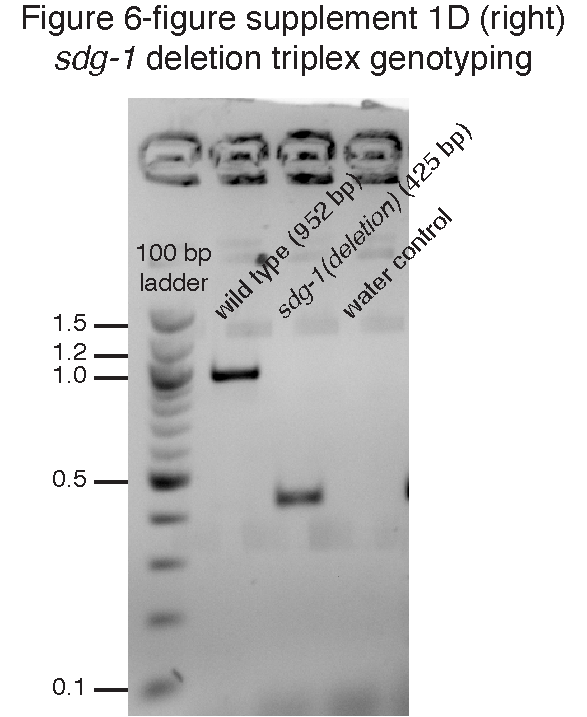

Supplement: Figure 6—figure supplement 1—source data 1. — Raw images are available at https://doi.org/10.6084/m9.figshare.25036142.v1. [file elife-99149-fig6-figsupp1-data1.zip › Figure6_figure_supplement_1/8d/sdg1_del_genotyping.tif]
